# From resistance to readiness: faculty development as the key to AI literacy in public health

**DOI:** 10.3389/fpubh.2026.1794913

**Published:** 2026-03-05

**Authors:** José A. Acosta

**Affiliations:** Ponce Health Sciences University School of Medicine St. Louis Campus, St. Louis, MO, United States

**Keywords:** AI literacy, algorithms, education, faculty development, public health

## Introduction

In 2019, researchers discovered that a widely used algorithm, which influenced care decisions for an estimated 200 million patients annually, systematically underestimated the health needs of Black patients. Because Black patients historically spent less on healthcare due to access barriers and systemic inequities, the algorithm concluded they were healthier than equally sick white patients. Correcting this bias would have increased Black patient enrollment in care programs from 17.7 to 46.5% (1). Future public health practitioners must be equipped to detect, evaluate, and prevent precisely this kind of algorithmic failure.

The stakes extend beyond individual patient care: artificial intelligence (AI) systems now manage disease surveillance, resource allocation, health misinformation detection, and pandemic response. Even when a human-designed, instruction-driven system appears to deliver benefits, it may still reproduce inequities and shift the harms onto entire communities. Public trust in health institutions erodes, and health equity gaps widen. Public health practitioners must evaluate AI-driven surveillance systems, assess algorithmic fairness in resource allocation, and combat AI-generated health misinformation.

Meeting these broader stakes requires more than individual enthusiasm; it requires a workforce trained to evaluate, implement, and govern AI responsibly in real-world public health systems. However, many faculty tasked with building that workforce report limited preparedness and insufficient institutional support. A consistent gap exists across medical and public health education: students eagerly engage with AI literacy, yet faculty often express significant hesitation. For instance, even with clear applications in epidemiologic analyses, educators who doubt the reliability of LLMs may choose not to incorporate them into instruction, limiting students' exposure and skills. Cultivating this balance of efficiency and critical oversight defines the AI literacy that public health now requires.

Faculty resistance and unpreparedness are among the primary rate-limiting steps in integrating AI literacy. Without addressing this bottleneck, curriculum reform will stall regardless of how competency frameworks are designed. This commentary synthesizes empirical and conceptual literature to address that implementation gap and to inform practical strategies for educators. It draws on multiple forms of evidence and interpretation, with a summary classification table provided at the end. Because evidence on public health-specific AI competencies remains limited, I draw primarily on related health professions literature. The next section synthesizes this literature to clarify the barriers and opportunities shaping AI literacy integration.

## Summary of the evidence

A targeted literature search was conducted in PubMed and Google Scholar using terms such as “generative AI,” “faculty development,” “health professions education,” “public health curriculum,” and “AI literacy.” Given the nascent nature of this field, the search prioritized public health-specific empirical studies when available, health professions education literature with transferable findings, and higher education AI adoption research to contextualize broader patterns. Theoretical frameworks from faculty development and change management literature informed the interpretation of these findings.

These searches revealed notable barriers to AI adoption among faculty. Across higher education, faculty describe recurring barriers to AI integration, including inadequate training, confidence gaps, academic integrity worries, and a lack of institutional guidance. Evidence specific to public health remains limited but informative. In a survey of 62 public health instructors, 48% reported using ChatGPT (OpenAI), while concerns about impaired learning (32%) and unethical use (11%) were common (2). As one instructor noted: “I've heard the accuracy depends on prompts; we need to consider training students (and faculty) how to best use prompts.” Similarly, another study found only 40% of respondents had tried ChatGPT, with trainees more likely than faculty to have done so. Beyond low adoption rates, significant implementation challenges compound this hesitancy. Concerns about bias, privacy risks, lack of transparency, and fear of job displacement fuel skepticism and further slow integration (3).

Broader higher education and health professions literature confirms these patterns. Educators report lacking confidence and competence to address generative AI effectively (4). Other studies identify practical obstacles: not knowing how to use tools, limited time to experiment, and unclear benefits (5). Emotional barriers also emerge, with technology-related distress and fear of obsolescence pointing to the need for humanizing AI training (6). A recent systematic review underscores the core problem: the scarcity of formal training, guidelines, and policy frameworks limits effective integration (7).

However, resistance is not universal. Recent evidence suggests a growing number of higher education faculty are engaging with AI tools. A 2025 Digital Education Council survey found that 61% of faculty worldwide reported using AI in their teaching practice, representing substantial increase from prior years (8). Faculty who receive structured AI training report marked increases in confidence and more favorable attitudes toward integration, suggesting that targeted interventions can shift the trajectory (9, 10). These findings suggest that faculty concerns, while valid, are addressable through appropriate institutional support and training. However, while these general faculty development approaches are necessary, they are insufficient for public health education. The discipline faces a distinct set of challenges that standard AI literacy frameworks do not address, requiring domain-specific competencies that extend beyond individual classroom applications.

## Public health's unique challenge

In addition to the barriers described above, public health faces further challenges tied to its mission. Specifically, it requires competencies that many clinical AI frameworks do not address, including population-level algorithm evaluation, health equity assessment, and governance of systems that affect communities. Public health practitioners must interpret AI outputs not only for individual patients but also for populations, asking whether algorithms exacerbate or reduce health disparities, distort surveillance data, or misallocate scarce resources.

The need for AI competencies aligns with broader public health workforce development priorities. The Council on Linkages Core Competencies for Public Health Professionals (2021) emphasizes critical analysis and evidence-based decision-making, skills directly applicable to AI evaluation (11). The Association of Schools and Programs of Public Health Leadership Competency Mapping initiative (2022–2024), funded by the Centers for Disease Control and Prevention, identifies emerging workforce training needs that increasingly include digital literacy and data science capabilities (12). Despite these established workforce development priorities, empirical evidence on public health faculty perceptions of generative AI remains limited (2, 13).

Generative AI shows immense promise for public health pedagogy but also presents inherent limitations and ethical considerations that require caution (14). Public health professionals must learn to assess AI outputs critically to ensure equitable and effective outcomes (15). Yet no validated competency framework currently exists for population health contexts. These population-level demands highlight why the absence of validated frameworks is so consequential: graduates will confront surveillance systems, misinformation campaigns, and resource-allocation tools that can perpetuate—or mitigate—inequities at scale.

The global record of algorithmic failures in public health illustrates what is at stake when these competencies are missing, particularly in resource-limited settings (16). Populations in low- and middle-income countries are especially susceptible to unfair outcomes from AI and machine learning (ML) systems, as these tools may reinforce socioeconomic disparities or political marginalization without adequate safeguards. A key case study from Pune, India, applied logistic regression models to questionnaire and peak flow data for pulmonary disease screening, such as chronic obstructive pulmonary disease (COPD). Systematic bias emerged in COPD prediction due to underlying differences in smoking prevalence (55 percent in men vs. 0 percent in women, varying by socioeconomic status), resulting in higher model accuracy for women than men and for high-socioeconomic-status vs. low-socioeconomic-status groups (16).

Another relevant analysis examined supervised machine learning models trained on the Indian Liver Patient Dataset for liver disease prediction (17). It identified significant sex bias, with female patients experiencing higher false negative rates across classifiers, leading to increased rates of missed diagnoses and potentially worsened outcomes for women in this context. These examples underscore the urgent need for public health schools to teach AI competencies, including bias evaluation and fairness-aware design in population-level systems, to prepare graduates for equitable governance of AI in diverse, resource-limited settings. The central question, then, is how can institutions move faculty from understandable resistance toward readiness to lead this work. Answering this question requires drawing on established frameworks from health professions education and change management, adapted to address public health's distinct challenges.

## From resistance to readiness

Health professions education offers effective strategies for institutional change. However, directly applying these frameworks for public health is insufficient without modifications that address unique population-level, equity, and governance concerns. Established change management and adult learning theories provide a robust structure for navigating these patterns. For instance, Rogers' Diffusion of Innovations framework categorizes adopters from innovators to laggards, explaining the spectrum between faculty who embrace AI and those who resist it (18). Complementing this, Hall's Concerns-Based Adoption Model conceptualizes a developmental shift in faculty concerns from self-focused issues, to management of tasks and logistics, and, ultimately, to the impact of the innovation on students (19). Finally, Knowles' principles of andragogy dictate that faculty development must treat educators as self-directed adult learners motivated by immediate professional relevance (20).

These principles of individual readiness and adult learning provide the necessary foundation for implementing specific curricular models. Building on that foundation, one such model—a recently proposed five-pillar framework for AI literacy integration—emphasizes technical foundations, ethical and regulatory literacy, experiential learning, governance and policy, and equity and access (21). However, realizing any such framework depends on the readiness of those responsible for delivering it, which in turn hinges on deliberate institutional strategies to support faculty development rather than assuming individual faculty will adapt on their own.

Faculty development must become infrastructure, not optional enrichment. Faculty adoption of AI depends on institutional support: training, ethical guidelines, and clear policies (7). Evidence indicates that training may improve readiness. In one single-institution study, structured workshops increased teacher confidence with generative AI tools by 30%, but replication across diverse settings is needed (22). Yet technical training alone may be insufficient. Recent research suggests AI training must also be humanizing, addressing fears of obsolescence alongside technical competencies (6).

Kotter's change management framework provides a useful lens for implementation (23). It emphasizes creating a sense of urgency, building guiding coalitions, and embedding new approaches within institutional culture. For AI literacy integration, this means connecting faculty development to accreditation pressures and workforce demands, engaging early adopters as change champions, and embedding AI competencies into promotion and tenure expectations.

Clear institutional policies are essential. In one study, no instructors had AI policies in their courses, a gap students felt acutely (2). As one noted: “Without concrete policies on what usage is acceptable, it is difficult to feel comfortable using such technologies.” This discomfort points to a broader need. Multiple studies emphasize clear guidelines around privacy, ethics, and regulatory frameworks when embedding AI training, with some recommending “train-the-trainer” models and ongoing ethical training for both educators and students (15, 24).

Successfully implementing these strategies requires first acknowledging that faculty resistance reflects genuine concerns about academic integrity, accuracy, and professional preparedness. Rather than dismissing these concerns or forcing adoption, effective integration demands addressing both technical and emotional barriers through robust training, clear policies, and adequate resources. With this foundation established, the following three complementary tools synthesize the evidence into an actionable framework.

**Figure 1:**
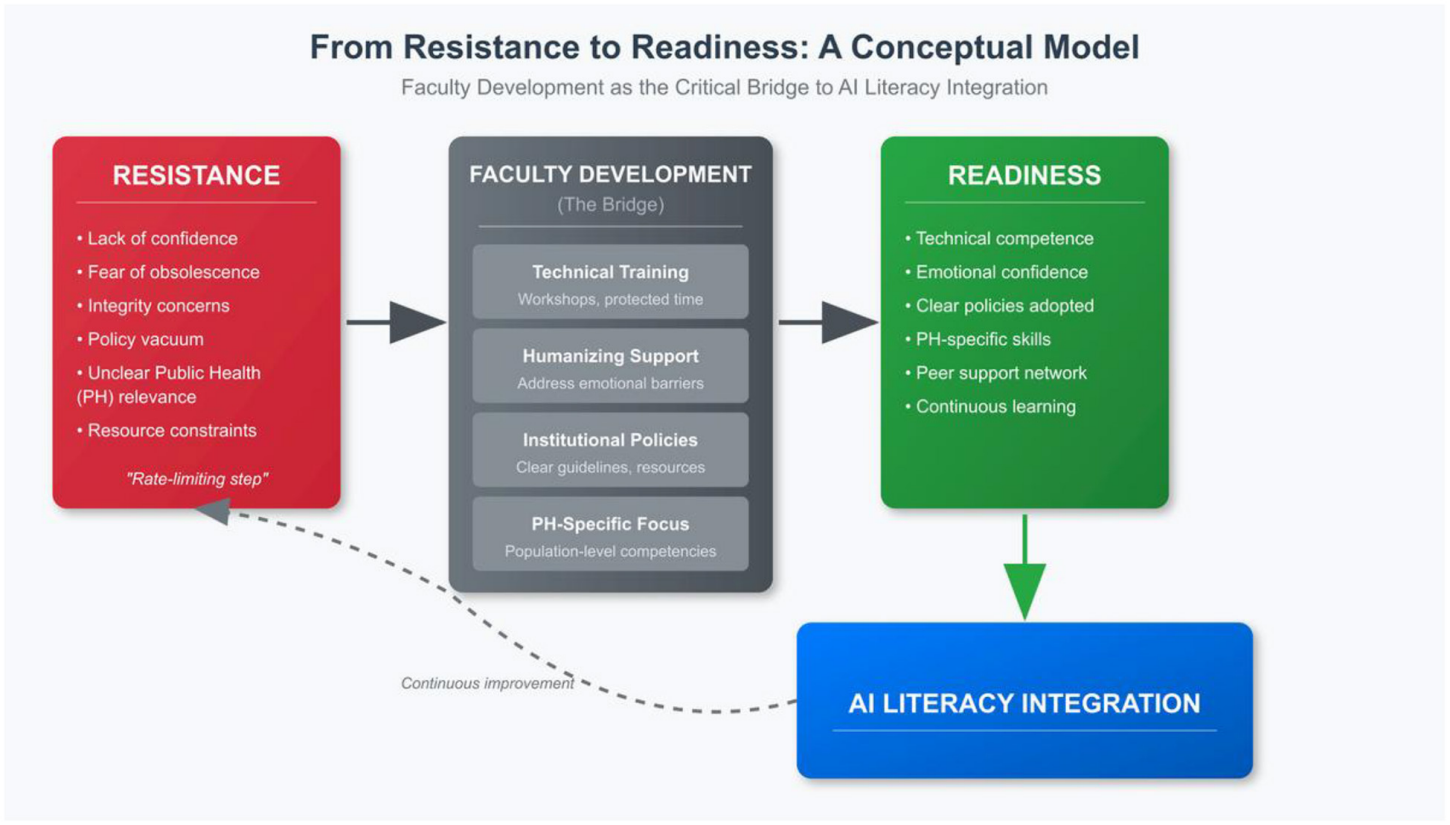
From resistance to readiness: a conceptual model of faculty development as the critical bridge to AI literacy integration in public health education.

Table 1 provides a comprehensive framework of strategies to overcome faculty barriers, organized by barrier type. Figure 1 presents a conceptual model illustrating the pathway from resistance to readiness. Box 1 offers a tiered implementation roadmap with short-term and long-term recommendations for institutional leaders, department chairs, and individual faculty. Together, these translate the theoretical frameworks and empirical evidence reviewed above into concrete levers for change at multiple levels of implementation.

**Table 1:** Evidence-based strategies for addressing faculty barriers to AI integration.

**Barrier**	**Authors**	**Level of evidence**	**Recommended strategies**	**Basis of recommendation**
Lack of training/ confidence	Moorhouse and Kohnke (4); Blanco et al. (5); Cordero et al. (22)	Empirical study (qualitative interviews) Empirical study (mixed-methods) Empirical study (survey)	• Structured faculty development workshops •Protected time for AI experimentation •Dedicated institutional resources for training •Peer mentorship programs	Evidence-based
Emotional barriers	Merkebu and Samuel (6)	Case study/innovation report	• Address fear of obsolescence directly •Humanizing training that validates affective concerns •Faculty support groups •Gradual integration approaches	Author synthesis
Academic integrity concerns	Anderson et al. (2)	Empirical study (cross-sectional survey)	• Redesign assessments to assume AI use •Focus on critical evaluation skills •AI-assisted but not AI-dependent assignments •Process documentation requirements	Evidence-based
Policy vacuum	Anderson et al. (2); Nikolic et al. (7)	Empirical study (cross-sectional survey) Systematic literature review	• Clear institutional guidelines •Syllabus templates with AI policies •Explicit expectations for acceptable use •Regular policy review and updates	Evidence-based
Public health-specific gaps	Conrad and Hall (14); Love et al. (15); Acosta (21)	Conceptual paper/descriptive case study Practice notes/conceptual paper Conceptual paper/perspective	• Develop population-level AI competencies •Address algorithmic equity in curricula •Surveillance system evaluation training •Community-level impact assessment skills	Expert consensus/best practice synthesis Author synthesis

Box 1 **Implications for institutions.**

**For deans and senior leadership**

***Short-term (0–12 months):***

- Allocate protected time for faculty AI exploration
- Issue clear institutional AI use policies
- Designate AI literacy point person or committee

***Long-term (1–3 years):***

- Integrate AI competencies into faculty evaluation criteria
- Establish sustainable funding for ongoing training
- Advocate for accreditation standards addressing AI

**For department chairs**

***Short-term (0–12 months):***

- Identify early adopter faculty as peer mentors
- Facilitate curriculum mapping to identify AI integration points
- Create psychologically safe spaces for experimentation

***Long-term (1–3 years):***

- Develop discipline-specific AI competency frameworks
- Build industry partnerships for real-world AI case studies
- Establish feedback loops for continuous improvement

**For individual faculty**

***Short-term (0–12 months):***

- Experiment with GenAI tools in low-stakes contexts
- Attend institutional workshops and seek peer mentorship
- Develop personal AI use policy for courses

***Long-term (1–3 years):***

- Integrate AI literacy learning objectives into courses
- Contribute to scholarship on AI in public health education
- Mentor colleagues transitioning to AI integration

These tools provide a foundation for institutional change. However, even well-designed implementation plans face substantial practical obstacles that warrant careful consideration.

## Discussion

Faculty resistance and the surrounding anxiety reflect genuine concerns regarding academic integrity, accuracy, and professional preparedness. Rather than forcing adoption, institutional leaders must create conditions for responsible integration by addressing both technical and emotional barriers through robust training, clear policies, and adequate resources. Implementation faces substantial obstacles, including constrained budgets and a lack of specific accreditation mandates for AI competencies. Because public health cannot govern what it does not understand, closing the AI literacy gap requires equipping the educators who train the next generation of practitioners.

This analysis has limitations. The empirical literature examining public health faculty perceptions of generative AI remains in its early stages, and much of this synthesis necessarily draws from broader health professions and higher education research. Generalizability to diverse public health contexts, such as community health centers or global health settings, requires further investigation. The rapid pace of AI advancement may eventually outstrip any static competency framework, underscoring the need for adaptive rather than fixed approaches. Despite these constraints, the risks of inaction are substantial.

The algorithm that systematically underestimated the health needs of Black patients was not discovered by an AI system; it was discovered by researchers who knew the right questions to ask. If we do not prepare faculty to teach the next generation how to ask those questions, we are not simply failing to keep pace with technology, we are failing the communities that public health exists to protect.
